# Transforming Breast Cancer Control in East Africa by Integrating Cytomorphology and Genetics Into National Policy

**DOI:** 10.7759/cureus.111147

**Published:** 2026-06-19

**Authors:** Josephine N Rioki, Mwangi Joseph, Rency Lel, Marshal Mweu, Lucy Muchiri

**Affiliations:** 1 Medical Laboratory Sciences, University of Nairobi, Nairobi, KEN; 2 Centre for Virus Research, HIV Department, Kenya Medical Research Institute, Nairobi, KEN; 3 Global and Public Health, University of Nairobi, Nairobi, KEN; 4 Human Pathology, University of Nairobi, Nairobi, KEN

**Keywords:** breast cancer control, breast cancer screening, cancer and genetics, cytomorphology, precision public health

## Abstract

The traditional approach to breast cancer control in Sub-Saharan Africa and East Africa has long been hindered by a reactive healthcare model that prioritizes symptomatic management over early, risk-stratified interventions. Drawing upon a multi-year research program conducted in Kenya that investigated cytomorphological patterns, epidemiological risk factors, and BRCA1/2 mutations, this perspective argues for a fundamental shift in national health policy. We propose that the current siloed diagnostic pathway may not be sufficient for the unique Kenyan and East African breast cancer phenotype, which is characterized by aggressive biology and early-onset presentation. This paper recommends repositioning fine-needle aspiration cytology (FNAC) as a triage tool followed by integration of genetic screening into the primary care framework. With this, East Africa can progressively move from a state of diagnostic delays to a model of precision public health.

## Editorial

The crisis of the palpable lump and the failure of reactive care

For decades, the global oncology community has emphasized mammographic screening as the gold standard for early detection, a position illustrated by a study by Naik et al. [1]. However, in the Kenyan context, where infrastructure is concentrated in urban referral centers and specialized imaging remains prohibitively expensive, the reality is that a palpable lump remains the primary entry point for diagnosis. Our research into cytomorphological patterns confirms a sobering reality: by the time a woman in Kenya identifies a lump and reaches a referral health facility, the cellular microenvironment often reflects advanced, high-grade malignancy [2]. Consequently, the current policy of general cancer awareness requires strengthening to address this gap. Awareness without a rapid, specialized diagnostic pathway leads to what may be termed diagnostic disillusionment, where patients present to the system only to encounter long delays for biopsies and histopathology. We contend that the health system must pivot from broad awareness to a rapid diagnostic triage system that treats every palpable lump as a potential molecular emergency.

Redefining fine-needle aspiration cytology as a diagnostic triaging tool

There is a recurring debate in modern oncology regarding the utility of fine-needle aspiration cytology (FNAC) versus core needle biopsy, as emphasized by Kim et al. [3]. In present-day breast oncology, core needle biopsy (CNB) is mostly desired over FNAC owing to its superior diagnostic accuracy and clinical utility. Beyond preserving tissue architecture and reducing inconclusive rates, CNB reliably differentiates between pre-invasive lesions and invasive carcinomas. Moreover, the intact tissue cores provide the required substrate for crucial immunohistochemical (IHC) profiling and advanced molecular characterization, which increasingly guides neoadjuvant and adjuvant treatment decisions. Whereas these clinical advantages make the CNB the standard of care in resource-replete settings, FNAC remains a crucial tool within the Kenyan referral system due to its rapidity, low cost, and minimal invasiveness.

In resource-replete settings, the latter is preferred for its ability to provide tissue architecture. However, in the Kenyan referral system, FNAC remains an indispensable tool due to its speed, low cost, and minimal invasiveness. Our perspective is that FNAC should be elevated from a preliminary test to a sophisticated gatekeeper tool. Our data show that specific cytomorphological features such as nuclear pleomorphism and necrosis are not just indicators of cancer; they are proxies for aggressive subtypes like triple-negative breast cancer (TNBC). Through training pathologists and cytologists to recognize these high-risk morphology patterns, the healthcare system can implement an emergency triage system. Classifying these features allows clinicians to fast-track high-risk patients for prioritized histopathological confirmation, followed by advanced molecular analysis and instant surgical consultation. This considerably reduces the delays typically associated with standard pathological queues. 

Genetic equity: the necessity of BRCA1/2 screening

Perhaps the most significant paradigm shift required in East African oncology is the mainstreaming of genetic testing. Policymakers often dismiss genetic screening for BRCA1 and BRCA2 mutations as a luxury reserved for high-income nations. Our research challenges this notion. The identification of significant pathogenic variants by Rioki et al. in Kenyan women with TNBC demonstrates that hereditary breast cancer is a clinically relevant reality in this population [4]. We argue for genetic equity, the principle that genetic risk assessment should be an integral part of public health strategies in Sub-Saharan Africa. When we identify a BRCA mutation in a symptomatic woman, we are not just diagnosing an individual; we are also identifying a high-risk family. In a region where families are large and multi-generational, a single genetic test has a multiplier effect, allowing for the prophylactic monitoring of sisters, daughters, and nieces. This is the ultimate form of early detection, preventing cancer before the palpable lump ever appears.

A roadmap for precision public health

To translate research findings into a national strategy, we propose a three-tiered precision triage roadmap that aligns with the Kenyan Ministry of Health’s goals for universal health coverage.

Tier 1: Epidemiological Risk Stratification

At the primary care level, health workers must move beyond simple clinical breast exams. They should utilize a standardized risk-assessment tool based on the epidemiological factors identified in our research, such as age at presentation, parity, and family history. Women who meet high-risk criteria should be given priority referral slips. This approach can further be decentralized to a community-driven public health strategy. This has the potential to transform breast cancer response by shifting the frontline from passive clinical waiting rooms directly into households of everyday citizens. For example, in Kenya and other countries, this can be achieved by leveraging community health promotors (CHPs), a formal cadre of community health workers embedded as trusted peers in community settings. They can be equipped with a localized breast cancer risk assessment tool. This tool can be digitized natively with the Electronic Community Health Information System (eCHIS), Kenya's national digital health platform, deployed as a mobile application.

In routine practice, CHPs utilize eCHIS on government-issued smartphones during door-to-door household visits. This app guides the CHPs through standard digital screening workflows, tracks patients' health longitudinally over time, and automatically synchronizes this community-level data with the national health database to facilitate timely facility referrals [5]. Although CHPs are non-specialist volunteers without formal medical degrees, in practice, the mobile app handles the clinical complexity behind the scenes. As they conduct household visits, the app prompts them with simplified dropdown questions to capture relevant epidemiological data such as age of early onset, parity, and family history, effectively turning CHPs into a crucial conduit for targeted early identification and grassroots health education without requiring immediate clinical examinations.

The digitized risk tool would then automatically trigger synchronized SMS alerts and dashboard updates that connect high-risk patients directly to regional tier 2 clinics for examination and further care. This handover protocol guarantees that the vulnerable women are actively navigated through the healthcare continuum, drastically reducing the time to diagnosis window and saving lives through precise intervention. This would transform breast cancer screening in Kenya, and many other countries, where breast cancer screening relies on irregular manual campaigns focused on clinical breast exams (CBE).

Furthermore, the development, integration, and use of artificial intelligence (AI) tools in the form of an algorithm designed for a digital cytology framework can greatly improve the diagnostic utility of FNAC by providing valuable information about breast cancer aggressiveness. Utilization of deep learning methods in the analysis of digitized cytological smear images can help accurately predict the phenotype of the malignancy through assessment of complex nuclear details, including nuclear pleomorphism, dense chromatin, and pronounced nucleoli. In a limited resource environment like that of the East African region, such an AI-based cytological test will, in this regard, enable rapid point-of-care prognostic classification. This, in turn, allows clinicians to immediately identify and fast-track patients with highly aggressive phenotypes for prioritized histopathological confirmation and expedited systemic therapy.

Tier 2: Rapid Cytomorphological Triage

In this approach, referral hospitals could establish one-stop Clinics where FNAC procedures will take place and results will be analyzed within a matter of hours. If the cytomorphology indicates high-grade malignancy, the patient can be immediately enrolled in the precision pathway. Implementation of this model has the potential to provide low- or middle-income countries (LMICs) with a scalable framework that could significantly contract the diagnostic window, moving from typical delays of several months towards a targeted single-day process for initial cytology triage. Eventually, such streamlined pathways are anticipated to improve survival rates for aggressive phenotypes by dramatically accelerating the time to definitive treatment. 

This approach addresses systemic LMIC infrastructure gaps through two crucial interconnected pillars. First, the model overcomes specialist shortage through the integration of telecytology. Clinics can leverage rapid on-site evaluation (ROSE), where local technologists prepare slides and upload high-resolution digital images to cloud-based networks. This allows for immediate off-site expert review, bringing specialist level diagnostics accuracy to under-resourced areas in real time. Second, the model mitigates patient loss through a strategy of immediate enrollment. To eliminate diagnostic dropouts, clinics implement a swift reflex protocol. If initial testing confirms malignancy, a reflex biopsy is performed on the spot. The patient is then instantly assigned a dedicated patient navigator who secures advanced staging and molecular workups before the patient leaves the facility, completely bypassing standard outpatient queues and securing a definitive path to treatment.

Tier 3: Reflex Molecular and Genetic Testing

To streamline care and reduce the testing burden and facilitate delivery of patient care, this tier sets forth a reflex molecular genetic testing process in all patients diagnosed with TNBC or tumors showing high-grade malignant features under cytological examination. In accordance with this protocol, after having conducted standard pre-testing genetic counseling and obtaining the patient's consent at his or her first clinical consultation, confirmation of a case meeting the established criteria will result in immediate BRCA1/2 mutation analysis without requiring further physician orders. Expanding this reflexive pathway to all high-grade malignancy cases, as determined by oncology rather than being confined to only those confirmed as TNBCs, is informed by local realities and epidemiological trends in Sub-Saharan Africa.

In Kenya's healthcare settings, confirmatory testing of TNBC diagnosis through histopathological and immunohistochemistry techniques can be a long, drawn-out process. High-grade morphology has been shown to have a significant association with aggressive biological behavior and a high prevalence of hereditary breast cancers among younger women in Africa. Using this parameter, therefore, provides a reliable and immediate way of identifying high-risk patients requiring targeted interventions. These clinical and structural aspects apply in the broader East region too. Member countries share a similar health background, characterized by a high burden of early-onset, biologically aggressive breast cancer. There are also cross-border health-seeking behaviors to access specialized oncology services, a practice increasingly supported by regional bodies such as the East Africa Health Research Commission (EAHRC), which aims to harmonize healthcare delivery and research policies across member states 

To ensure affordability and minimize capital expenditure, this approach leverages and repurposes the robust, regional HIV and COVID-19 PCR infrastructure and bio-banking networks for oncology assays and next-generation sequencing. This scale-up strategy is equally viable across East Africa's shared global health infrastructure. For long-term equity and fiscal sustainability, these panels require integration into available health insurance fund reimbursement under universal health coverage. This is possible, for example, in Kenya, under the Social Health Insurance Fund (SHIF). Whereas the earlier insurance system, National Health Insurance Fund (NHIF), was less accommodating to such provisions, the current SHIF, as well as Emergency, and Chronic and Critical illness fund (ECCIF), have increased the annual benefit amount per individual patient for cancers to KSH 800,000 (USD 6,184.29).

This system covers both advanced diagnosis and the comprehensive coverage of costly breast cancer treatment without any out-of-pocket expense. This is not to say there are no other costs incurred, but this indicates the potential to expand diagnostic and treatment insurance coverage. In light of this, the discovery of genetic mutations can lead to effective cascade testing for family members concerned. Identification of positive mutations, therefore, would trigger cascade screening for relatives, effectively shifting oncology towards proactive, preventive, and precision-based surveillance.

Conclusions

The evidence from our study of Kenyan breast pathology is clear: the disease we are fighting is biologically aggressive and genetically driven. We can no longer afford to manage breast cancer through the lens of late-stage symptomatic care. This perspective calls upon the Ministry of Health, academic institutions, and international partners to endorse a diagnostic model that is as aggressive as the disease itself. By integrating the microscopic (cytology) with the molecular (genetics), Kenya can lead the region in demonstrating that precision oncology is not a luxury, but a necessity for the global South. Through cross-border health cooperation and regional policy alignment, this framework offers a scalable outline for neighboring nations facing identical epidemiological and health realities.

## References

[1] Naik S, Varghese AP, Asrar Ul Haq Andrabi S, Tivaskar S, Luharia A, Mishra GV (2024). Addressing global gaps in mammography screening for improved breast cancer detection: a review of the literature. Cureus.

[2] Rioki JN, Mweu M, Rogena E, Songok EM, Mwangi J, Muchiri L (2025). Factors associated with breast lesions among women attending select teaching and referral health facilities in Kenya: a cross-sectional study. PLoS One.

[3] Kim A, Lee HJ, Kim JY (2025). Breast fine-needle aspiration cytology in the era of core-needle biopsy: what is its role?. J Pathol Transl Med.

[4] Rioki JN, Muchiri L, Mweu M (2023). BRCA1 and BRCA2 mutations and their clinical relevance in selected women diagnosed with triple-negative breast cancer in Kenya: a descriptive cross-sectional study. Pan Afr Med J.

[5] Ireri S, Waiganjo P, Ochieng DO (2025). An exploration of the successful scale-up of the electronic community health information system in Kenya. Oxf Open Digit Health.

